# Acceptance of Virtual Reality Simulation Training for Stoma Care by Healthcare Providers: A Pilot Questionnaire Study After Viewing Prototype Imagings

**DOI:** 10.7759/cureus.65465

**Published:** 2024-07-26

**Authors:** Kengo Kai, Hiromi Shinoda, Emi Takeiri, Takeomi Hamada, Mayumi Chikubu, Yuko Kodama, Kazuhiro Higuchi, Atsushi Nanashima

**Affiliations:** 1 Department of Surgery, University of Miyazaki, Miyazaki, JPN; 2 Department of Nursing, University of Miyazaki Hospital, Miyazaki, JPN

**Keywords:** education, virtual reality, healthcare providers, ostomate, stoma care

## Abstract

Objectives: Educational simulation systems using virtual reality (VR) with head-mounted devices are spreading into the medical field. We developed an innovative training system whereby experienced ostomates can share their proficient stoma self-care techniques with novice ostomates through VR simulations, enabling anytime, anywhere learning. We examined the questionnaire study to assess the acceptance of VR simulation training for stoma care by healthcare providers.

Methods: This study was conducted for the participants, nurses, and doctors, at the 39th Kyushu Stoma Rehabilitation Research Meeting with the organizer’s permission. We created two VR simulation prototypes from the perspective of ostomates and caregivers using modeled stomas. We conducted a qualitative study through a questionnaire with healthcare professionals regarding their experiences of viewing VR videos.

Results: The study included 20 (52.6%) nurses certified in wound, ostomy, and continence (WOC) care, 16 (42.1%) non-WOC nurses, and two (5.3%) medical doctors. Over 90% of participants showed a positive inclination towards the practical application of the system in clinical settings for both scenarios. A significantly higher number of nurses in the non-WOC nurse group expressed a definite interest in using the imaging from the ostomate’s perspective versus that in the WOC nurse group (81.3% vs. 40%, P=0.013).

Conclusions: From this survey, we concluded that the respondents felt a VR training system was a positive experience, to say it is acceptable means that it was as good as the standard of care. ​​​​​​Particularly, non-WOC nurses, with fewer opportunities in stoma care compared to WOC nurses, showed stronger interest in practically implementing this innovative training system.

## Introduction

Stoma refers to a surgically created opening in the abdominal wall, typically formed when colonic excretion becomes challenging due to conditions such as cancer or inflammatory bowel disease, that often necessitates partial or complete colon resection or intestinal obstruction. In Japan, the population of individuals with stomas, known as “ostomates”, is estimated to exceed 210,000, with further growth anticipated in the future, according to the regular statistical surveys conducted by Japan's Ministry of Health, Labour and Welfare [1]. Ostomates encounter various personal and social challenges [2,3], including acquiring skills for stoma self-care and coping with psychological issues associated with reintegrating into society. Instruction in stoma self-care typically begins during the hospitalization period following stoma construction surgery. Early acquisition of these care skills is crucial for fostering confidence in social reintegration [4]. However, in Japan, due to revisions in medical reimbursement policies, there is a need to shorten the average length of hospital stays. This limitation on hospitalization time restricts the opportunity for ostomates to acquire necessary self-care skills and develop a comprehensive understanding of daily life with a stoma. Consequently, many ostomates transition to home care with feelings of anxiety highlighting the importance of enhancing technical and psychological support from healthcare providers [5].

Educational simulation systems utilizing virtual reality (VR) with head-mounted devices have shown promising advancements within the medical field, including emergency medicine, surgery, nursing, and various other healthcare disciplines. VR offers immersive experiential learning and simulating on-site experiences that contrast with traditional passive two-dimensional (2D) instructional materials [6,7]. Moreover, this video technology has the potential to foster empathy through simulated experiences, transcending geographical and time limitations, thus making it a vital tool for individuals with internal disabilities seeking psychological connections.

On the basis of this background, we have developed a stoma self-care training method for ostomates utilizing VR technology, currently under patent application, and launched it as the “OSTrain-VR Project”. The project team aims to create an innovative simulation system whereby experienced ostomates can share their proficient stoma self-care techniques with novices through VR imagery, enabling them to engage in simulations at their convenience and as often as needed. Similarly, the expert care techniques of experienced nurses can be shared as immersive experiences for ostomates’ families, less experienced healthcare providers, and medical students. Departing from the conventional approach of healthcare professionals leading ostomate guidance, this sharing system, rooted in ostomates’ or caregivers’ subjective experiences, endeavors to provide not only technical learning outcomes but also psychological support such as empathy. It aims to achieve both cognitive learning effects and to nurture connections and warmth among individuals concurrently.

To translate this concept into practical implementation, it is essential to directly consult healthcare providers actively engaged in stoma care to gather their insights regarding its application in real medical environments. To achieve this objective, we developed two prototypes of VR simulation imaging that capture the perspectives of both ostomates and caregivers using modeled stomas. Subsequently, we conducted a questionnaire survey among healthcare professionals working in various facilities to solicit their feedback.

## Materials and methods

Study design and participants

To pursue this innovative training approach for ostomates and caregivers, we collaborated with individuals from the University of Miyazaki Hospital, the Miyazaki prefectural branch of the Japan Ostomy Association, and L.A.B Inc. (Technologies Building, 75 Tashiro-cho, Miyazaki City, Miyazaki Prefecture, 880-0855, Japan), each contributing diverse ideas. This collaborative team, named the OSTrain-VR Project Team, consists of certified nurses in wound, ostomy, and continence nursing (WOC nurses), surgeons, experienced ostomates, and VR imaging developers. This collaboration has enabled us to create educational simulation imaging that is not only medically accurate but also emotionally impactful and visually striking. We showcased this VR system at the 39th Kyushu Stoma Rehabilitation Research Meeting held in Kagoshima City on July 29, 2023, with the organizer’s approval. Following the VR video presentations, a questionnaire survey was administered to the participants, including nurses and doctors involved in stoma care. This study was approved by the Institutional Ethics Committee of the University of Miyazaki (study number: O-1476). All participants included in this study provided consent via an opt-out format.

VR simulation video for ostomates and caregivers

**Figure 1:**
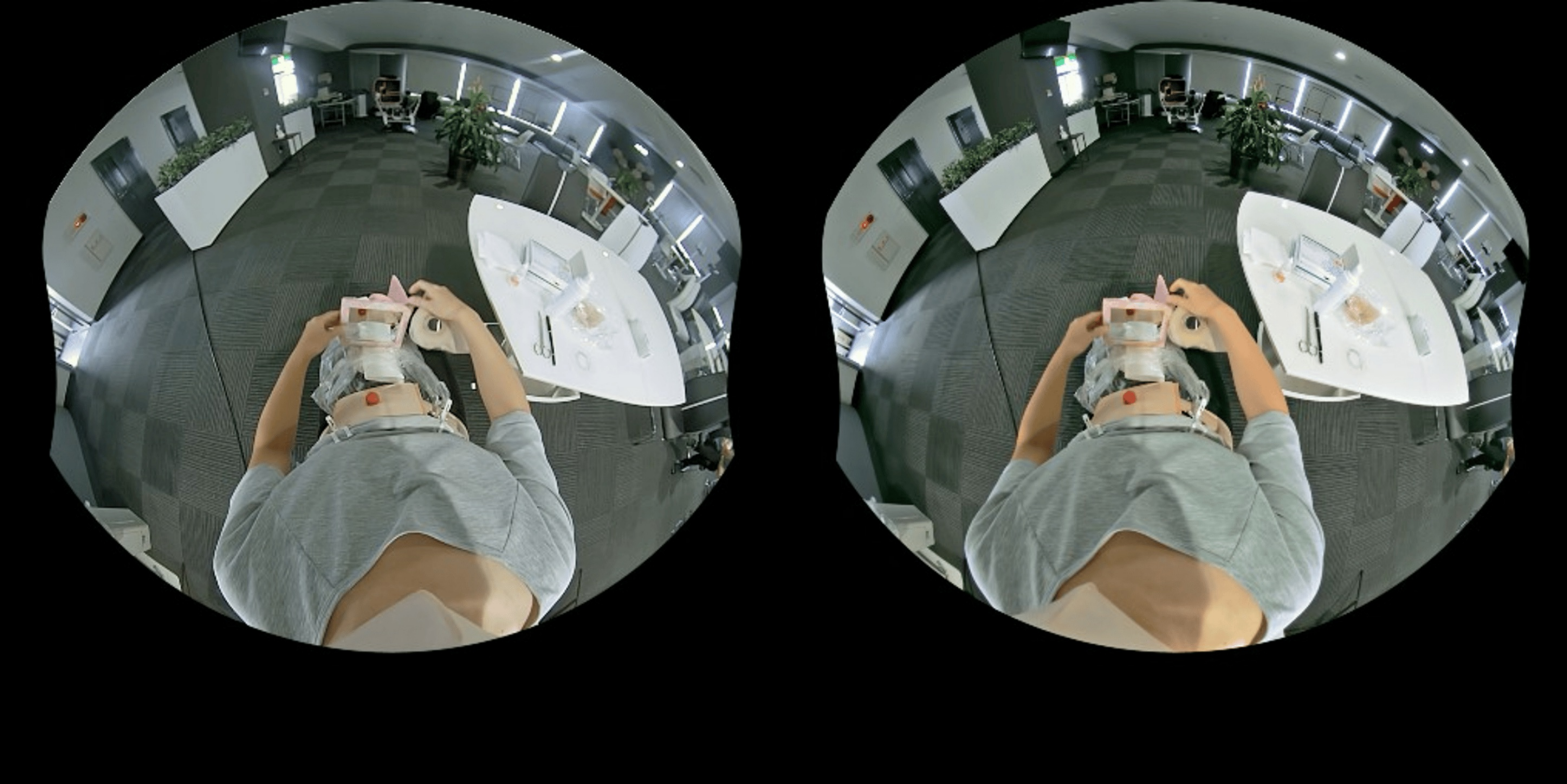
The virtual reality educational video was filmed from an ostomate’s perspective using a simulated stoma. The subject is using a hand mirror to check the skin condition in the hard-to-see area below the stoma.

**Figure 2:**
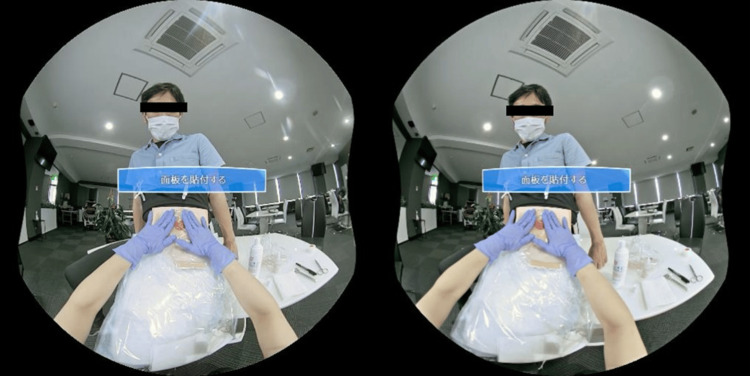
The virtual reality educational video was filmed from a caregiver’s perspective using a simulated stoma. The caregiver can experience stoma care while following instructions. Japanese caption explains "apply the stoma wafer".

We created two scenarios for VR simulation videos: (1) stoma self-care from the ostomate’s perspective (Figure 1) and (2) guided stoma care from the caregiver’s perspective (Figure 2). Approximately three-minute-long VR images were filmed and edited to depict the stoma care process, including stoma pouch detachment, skin cleansing, and pouch application, from each perspective. The videos utilized a healthy individual wearing a model stoma belt as the subject. This decision was made to ensure that detailed shooting conditions, such as experiential position, hand positioning, and camera distance from the stoma, were captured accurately. Verification of effectiveness and validity using a model was deemed necessary before filming involving actual ostomates commenced. The video content was shot using a VR-dedicated camera system, including the LUMIX GH5S (Panasonic, Osaka, Japan) and Insta360 Pro 2 (Insta360, Shenzhen, Guangdong, China). Explanatory captions and audio guides were added to provide clear insights under the supervision of WOC nurses and ostomates.

This VR training system offers a broad 180-degree perspective, providing opportunities to present information in ways not achievable with traditional filming methods. The simulations were displayed on a DPVR-4D® Pro (Shanghai, China), a 4K compatible head-mounted display licensed by L.A.B Inc., allowing participants to freely turn their head and body in any direction to gather relevant information, similar to being present in the actual location. Observant participants could notice subtle details, such as their surroundings, home conditions, or other concurrent activities in the space. Presenting the content in cine-VR encourages active viewing, where viewers can choose what to watch and pay attention to, thus increasing immersion and stimulating intellectual and emotional engagement. Viewers experience a sense of accomplishment as they notice subtle details planted by the filmmaking team to enhance the overall experience.

Questionnaire

The survey questionnaire consisted of six questions related to the VR training images provided by the OSTrain-VR Project (Table 1).

**Table 1:** The questionnaire related to the VR training images provided by the OSTrain-VR Project. VR: virtual reality

Questions for participants
Q1. What is your occupation? (Nurses were asked to specify whether they were WOC nurses or non-WOC nurses.)
Q2. Have you tried other video content that utilizes VR?
Q3. Did you feel inclined to use VR content for stoma care education from the perspective of an ostomate in clinical practice? Please choose from the following options: (“Definitely want to use”, “Want to use with free access”, “Difficult to use”)
Q4. Did you feel inclined to use VR content for stoma care education from the perspective of a caregiver in clinical practice? Please choose from the following options: (“Definitely want to use”, “Want to use with free access”, “Difficult to use”)
Q5. Were there any physical issues or challenges encountered while using VR content?
Q6. Were there any technical issues or challenges encountered while using VR content?

Statistical analysis

All categorical variables were summarized as numbers and percentages and were compared between groups using Fisher’s exact test or the chi-squared test, as appropriate. Statistical analyses were performed using StatFlex version 7 (Artech, Osaka, Japan), and P-values of <0.05 were considered statistically significant.

## Results

In total, 38 individuals consented to participate in the study, and all of them (100%) responded to our questionnaire after watching the stoma care education imaging with the VR system. Among the participants, 36 (94.7%) were nurses, with 20 (52.6%) being WOC nurses and 16 (42.1%) being non-WOC nurses. Only two participants (5.3%) were medical doctors (Table 2). The majority of participants (71.6%) had not been exposed to other VR imaging contents before this study.

**Table 2:** Demographic characteristics of the participants VR: virtual reality; WOC nurse: nurse certified in wound, ostomy, and continence nursing

Characteristics	Participants (N=38)
Occupation, n (%)	
WOC nurse	20 (52.6)
Non-WOC nurse	16 (42.1)
Medical doctor	2 (5.3)
History of watching VR videos	
Yes	7 (18.4)
No	31 (71.6)

Acceptance of VR video education: ostomate’s perspective

Overall, 22 participants (57.9%) responded with “Definitely want to use” in clinical practice, whereas 14 (36.8%) responded with “Want to use with free access” (Table 3). Reasons given for wanting to incorporate this technology included the usefulness of ostomates grasping the imagery in the preoperative phase, and the ability of caregivers to experience patient-oriented self-care from the patient’s perspective through VR, thus fostering heightened awareness of providing empathetic nursing care. Two respondents (5.3%), who answered with “Difficult to use”, expressed concerns about technological challenges that would lead to confusion among the elderly. Interestingly, when stratified by WOC nurses (n=20) and non-WOC nurses (n=16), the rate of those responding with “Definitely want to use” was significantly higher in the non-WOC nurse group compared to the WOC nurse group (81.3% vs. 40%: P = 0.013).

**Table 3:** Acceptance of virtual reality video education: Ostomate’s perspective WOC: wound, ostomy, and continence nurse

	Overall	WOC	Non-WOC	P-value
	(N=38)	(N=20)	(N=16)	
Acceptance, n (%)				
“Definitely want to use”	22 (57.9)	8 (40)	13 (81.3)	0.013
“Want to use with free access”	14 (36.8)	10 (50)	3 (18.7)	0.052
“Don’t want to use”	2 (5.3)	2 (10)	0	

Acceptance of VR video education: caregiver’s perspective

In total, 19 participants (50%) responded with “Definitely want to use” in clinical practice, whereas 16 (42.1%) responded with “Want to use with free access” (Table 4). Reasons given for wanting to incorporate the technology included “being able to supplement the lack of experience in non-specialized nurses” and “being effective in practically educating supportive family members”. Three respondents (7.9%) who answered “Difficult to use” cited “not perceiving advantages compared to existing 2D images” as their reason. No significant difference was observed in this response between the WOC group and the non-WOC group.

**Table 4:** Acceptance of virtual reality video education: Caregiver’s perspective WOC: wound, ostomy, and continence nurse

	Overall	WOC	Non-WOC	P-value
	(N=38)	(N=20)	(N=16)	
Acceptance, n (%)				
“Definitely want to use”	19 (50)	8 (40)	10 (62.5)	0.18
“Want to use with free access”	16 (42.1)	11 (50)	4 (25)	0.07
“Don’t want to use”	3 (7.9)	1 (10)	2 (12.5)	0.418

Physical and technical tissues while watching VR videos

All participants were able to watch both of the approximately three-minute-long VR videos to the end, but four individuals (10.5%) reported experiencing motion sickness, and two (5.3%) reported mild dizziness (Table 5). Those who were aware of their symptoms experienced rapid improvement. No participants reported technical issues such as with image resolution, lighting, or volume adjustments.

**Table 5:** Occurrence of physical and technical problems while watching virtual reality videos

Characteristics	Participants (N=38) n (%)
Physical problems, n (%)	
None	32 (84.2)
Motion sickness	4 (10.5)
Mild dizziness	2 (5.3)
Technical problems, n (%)	
None	38 (100)

## Discussion

VR technology has become extensively integrated across diverse realms of human activity, serving as a medium that “enhances the perception of environments, phenomena, and behaviors”. As contributions of VR to social life, the Tokyo Fire Department's VR disaster prevention experience vehicle offers immersive training through simulations of earthquakes, fires, and wind and water disasters. Additionally, there are reports of VR travel initiatives for individuals with physical disabilities and activity limitations. A defining characteristic of VR is its ability to create a sense of immersion, making individuals feel as though they are present in a virtual world [8]. Utilizing head-mounted displays, individuals can engage in activities that closely replicate real-world experiences. The recent improvements in the performance and affordability of VR devices have further accelerated its widespread adoption. Therefore, the current proliferation of this new technology in medical education is a natural progression.

When it comes to applying VR to surgical education, there is a practical application known as “Holoeyes®”, developed to utilize CT data for the extraction of organs in the form of 3D polygons [9]. This application enables its utilization as a preoperative simulation tool. Furthermore, Nanashima et al. [10] have undertaken initiatives to provide an educational experience not only for surgeons but also for medical students and surgical assistance nurses. They utilize a VR-dedicated camera to capture surgical footage from the perspective of the surgeon and the assistant, allowing observation of the same surgery from diverse viewpoints. In nursing education, Jung et al. [11] have reported on a VR-based training system for intravenous injection. Through the combined use of VR training and simulator-based training, a significant improvement in the accuracy of intravenous injections was observed. Various applications of VR have been reported, including resuscitation education for midwives [12] and the acquisition of skills in urinary catheter insertion [13].

Furthermore, VR is increasingly anticipated not only as a tool for educating healthcare professionals but also as a means to enhance treatment outcomes for patients. You et al. [14] conducted a randomized controlled trial targeting chronic stroke patients to assess a VR-based musculoskeletal rehabilitation program. In that study, a significant improvement in motor function was reported compared to conventional treatment, and reconstruction of the motor cortex was observed in magnetic resonance imaging (MRI) findings. Moreover, the use of VR for pain relief has also been reported. Hoffman and colleagues [15,16] concluded that immersing individuals in VR games allows them to divert attention away from pain. Using MRI, they reported a significant reduction in activity in the somatosensory cortex, insula, and thalamus corresponding to the painful body region.

In the present preliminary study, we explored the attitudes of healthcare providers toward a VR simulation training system for stoma care. The assessment involved hands-on sessions and questionnaire surveys conducted during a research conference focused on practical stoma care. We developed the prototype of VR simulations for self-care from the perspective of ostomates and caregiving from the viewpoint of caregivers. In both scenarios, over 90% of participants showed a positive inclination towards its practical application in clinical settings. However, the degree of enthusiasm was evenly divided between “definitely want to use” and “want to use with free access”. A significant finding in this study highlighted that non-WOC nurses, whose opportunities to be involved in stoma care are limited compared to those of WOC nurses, showed a stronger interest in the practical implementation of this innovative training system.

However, our research has limitations, including a small participants' number, selection bias, and lack of a control group. As stated in the methods section, this study design was conducted with healthcare professionals who viewed the VR during the one-day Kyushu Stoma Rehabilitation Research Meeting, so we did not have control over the number of participants. Our findings may be susceptible to selection bias, as individuals who volunteered to participate may have been more willing or motivated to use a novel educational program about ostomates. Additionally, we acknowledge that a three-minute short VR presentation may be too brief for explaining scientific products. However, this study was conducted as a pilot study with safety as the top priority. Based on empirical advice from the VR engineering team, we decided to produce videos of three minutes in length to allow time to address potential issues such as discomfort or headset misalignment. This point has been added to the limitations section of the discussion.

This study did not include a 2D-video program as a comparison group. We believe that 2D video and VR are not necessarily mutually exclusive. While VR provides a sense of immersion and empathy that 2D video cannot offer, 2D video can provide personalization by using smartphones to capture patient-specific educational content. To evaluate the effectiveness of VR and 2D video, it will be necessary for care providers to view videos through VR, 2D, and both interventions.

Moving forward with our project, we aim to develop VR videos featuring actual ostomates, integrating the insights gained from the prototype video production and feedback received from healthcare professionals who reviewed the prototypes in this study. Our next step involves conducting a prospective clinical study to implement VR-based training protocols for patients undergoing stoma construction surgery. This research endeavor seeks to analyze and assess not only the technical and psychological advantages experienced by ostomates through the device but also the technical hurdles encountered by healthcare professionals when utilizing the VR system in real-world scenarios.

## Conclusions

Patients and healthcare providers need ongoing and repeated training to help them improve and maintain their knowledge. Our findings support a VR training system as an acceptable approach for ostomates and their caregivers. Particularly, non-WOC nurses, with fewer opportunities in stoma care compared to WOC nurses, showed stronger interest in implementing this innovative training system practically. We aspire to further substantiate the potential influence of this innovative technology on individuals with stomas through future research endeavors.
